# Comparison between Additive and Subtractive CAD-CAM Technique to Produce Orthognathic Surgical Splints: A Personalized Approach

**DOI:** 10.3390/jpm10040273

**Published:** 2020-12-11

**Authors:** Giuseppe Palazzo, Vincenzo Ronsivalle, Giacomo Oteri, Antonino Lo Giudice, Corrado Toro, Paola Campagna, Romeo Patini, Salvatore Bocchieri, Alberto Bianchi, Gaetano Isola

**Affiliations:** 1 Department of General Surgery and Surgical-Medical Specialties, School of Dentistry, University of Catania, Via S. Sofia 78, 95124 Catania, Italy; gpalazzo@unict.it (G.P.); vincenzo.ronsivalle@hotmail.it (V.R.); nino.logiudice@gmail.com (A.L.G.); corradotoro@hotmail.com (C.T.); paolacampagna91@gmail.com (P.C.); salvo.bocchieri@gmail.com (S.B.); alberto.bianchi@unict.it (A.B.); 2 Department of Biomedical and Dental Sciences and Morphofunctional Imaging, School of Dentistry, University of Messina, Via Consolare Valeria 1, 98123 Messina, Italy; oterig@unime.it; 3 Fondazione Policlinico Universitario A. Gemelli IRCCS, Institute of Dentistry and Maxillofacial Surgery, Università Cattolica del Sacro Cuore, 00168 Rome, Italy

**Keywords:** milling technology, prototyping, 3D printing, splints, dental materials

## Abstract

The present study aimed to evaluate the accuracy of digitally designed surgical splints generated with milling technology (material subtractive procedure) and with 3D printing technology (material additive procedure) through a customized approach in the planning of surgical orthognathic splints. Cone beam computed tomography (CBCT) examinations and scanned dental models of 10 subjects who had required surgical treatment of skeletal malocclusion were included. Simulation of the orthognathic surgery was performed according to dento-skeletal and aesthetic characteristics of the subjects and the visual treatment objective (VTO), using Dolphin3D software (Dolphin Imaging, version 11.0, Chatsworth, CA, USA). Afterward, the Appliance Designer software (3Shape A/S, Copenhagen, Denmark) was used to digitally design the surgical splints that were generated twice using laser stereolithography technology (DWS 0.29D, DWS, Vicenza, Italy) and milling technology (Sirona inLab MC X5). Finally, each physical splint was digitalized using a desktop scanner (D500 3D, 3Shape A/S, Copenhagen, Denmark) in order to perform deviation analysis using the original project as a reference. The relative percentage of matching (trueness) was calculated (Geomagic Control X software (3D Systems, version 2018.1.1, 3D Systems, Rock Hill, SC, USA). An Independent Student’s *t*-test was used to statistically analyze the data. The milled splints showed a lower value of root to mean square (RMS) relative to the original project (0.20 mm ± 0.018) compared to the prototyped splints (0.31 ± 0.021) (*p* < 0.001). According to the present findings, surgical splints generated with milling technology present higher trueness compared with 3D printing technology.

## 1. Introduction

The success of orthognathic surgery therapy is achieved by obtaining a correct balance between occlusal relationship, facial harmony, and smile aesthetics [1]. A preoperative collection of patients’ records reporting quantitative and qualitative information on dento-skeletal and facial deformities is fundamental for diagnostic and therapeutic purposes [2]. In this regard, all preoperative patient data are acquired from different sources and methods, including clinical examination, dental casts, cephalometric exams, facial arches, articulator models, and patient photographs [2]. All these diagnostic records are important to define the final preoperative maxillo-mandibular relationship that would represent the final relation between both jaws after surgery. The preoperative phase of orthognathic surgery ends with the construction of the customized surgical splints; this appliance allows the surgeons to register the new virtual position of the jaw and to guide the surgical procedure predictably and according to the defined treatment plan [3].

Advances in 3D imaging technologies have led to the improvement of specific software for the planning of three-dimensional virtual orthognathic surgery (3D) [4] and for the generation of surgical splints by using computer-aided design and manufacturing (CAD/CAM) [4,5]. The main benefits of 3D planning in orthognathic surgery are the high-definition virtual reproduction of the skull and teeth, useful for the production of highly defined digital splints [6,7], which determines, in the surgical workflow, more accurate planning and better long-term results [7,8].

Previous studies have addressed their investigations to the accuracy of virtual surgical planning (VSP) based on a computer-assisted method that was found to improve the diagnostic and therapeutic preoperative treatment plan [9] and its combination with the orthodontic treatment [10]. However, limited research data are available on the accuracy of digitally designed occlusal appliances and on the surgical splints obtained with different CAD/CAM technologies. A recent study [11] investigated the fitting properties of prototyped occlusal splints designed with different offset parameters and suggested that further studies are required to test the accuracy of different splints realized with different materials and CAD/CAM technologies in order to provide more comprehensive information on this topic. In this regard, Rapid Prototyping (RP) and milling technology are mostly used to generate physical models. Scientific evidence [12] confirms that milling machining (subtractive method) is more specific and accurate, but there are still many difficulties in creating objects with sophisticated markers, whereas RP (additive method) can generate forms with sophisticated features, thus increasing the efficiency of the procedure.

The present study aimed to evaluate the accuracy of digitally designed surgical splints generated with milling technology (material subtractive procedure) and 3D printing technology (material additive procedure). We carried out the surface-to-surface digital technique and deviation analysis of the designed surgical appliances. The null hypothesis was the lack of statistically significant differences of the Euclidean distances obtained between the generated occlusal splints and the original digital models.

## 2. Materials and Methods

For the present ex-vivo investigation, we recruited diagnostic records of 10 subjects who had required plaster models and Cone beam computed tomography (CBCT) examinations for surgical treatment of skeletal malocclusion. All plaster models were recruited in private orthodontic practice in Catania, Italy, and were digitalized by using a desktop scanner (D500 3D, 3Shape A/S, Copenhagen, Denmark). The present study was carried out following the Helsinki Declaration and obtained positive response by the Institutional Review Board of Catania University (IRB protocol number: 119/2020/PO).

The Dolphin3D software (Dolphin Imaging, version 11.0, Chatsworth, CA, USA) was used to perform the superimposition between the digital models and the CBCT and to perform the simulation of the orthognathic surgery to correct the malocclusion. In this regard, the virtual surgical planning, including virtual model surgery and fabrication of splints, was performed according to the dento-skeletal and aesthetic characteristics of the subjects and the visual treatment objective (VTO).

**Figure 1:**
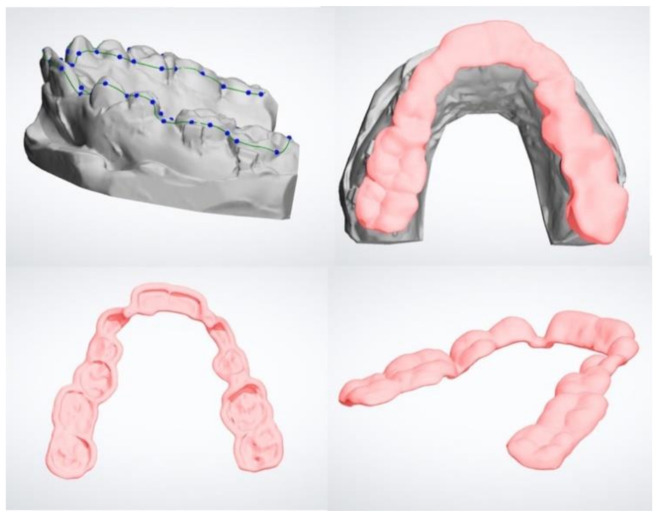
Digital design (CAD) of surgical splint: definition of spline curve and generation of 3D model on the upper arch.

**Figure 2:**
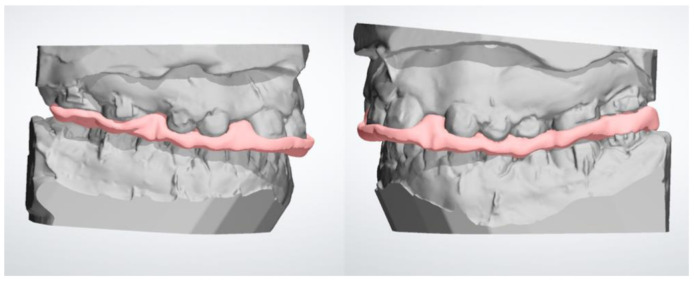
Digital design (CAD) of surgical splint: registration of the digital splint according to the new maxilla–mandibular relationship (surgery simulation).

The Stereo Lithography interface (*.stl*) files of maxillary and mandibular models with the new coordinate systems (i.e., after surgery simulation) were sent to a laboratory specializing in digital orthodontic manufacture in order to digitally design the occlusal splints using the Appliance Designer software (3Shape A/S, Copenhagen, Denmark). In particular, the appliance was designed with an offset (radial distance from the dental crown to the splint) of 50 microns and a radial thickness that allows contact between the lower arch and the appliance. The spline curve that was traced by an expert dental CAD operator surrounded the occlusal surfaces of the teeth, avoiding creating undercuts because the splint must be absolutely passive in the mouth and needs the minimum thickness to avoid contact with brackets (Figure 1). At the end of the designing of the device, a Boolean subtraction was performed between the CAD Michigan splint and the lower model; this operation allowed us to digitally dig out the cusps of the inferior teeth from the newly designed device, in order to obtain the surgical splint CAD file ready to be sent to a CAM machine (Figure 2). This file became the CAD reference model for deviation analysis. 

**Figure 3:**
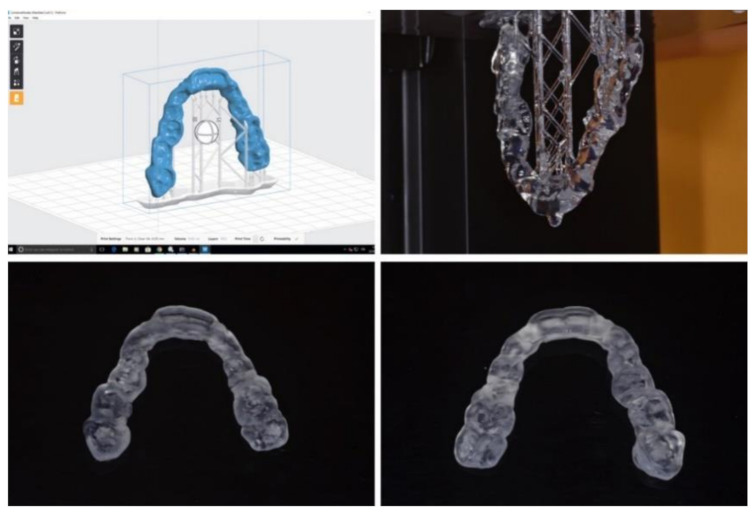
Prototypation (3D printing) of surgical splint.

**Figure 4:**
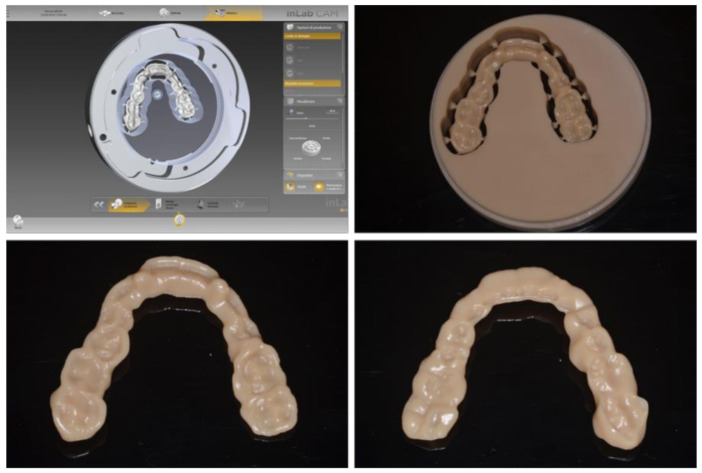
Surgical splint obtained from the milling process.

Afterward, each splint was manufactured by using (1) laser stereolithography technology (DWS 0.29D, DWS, Vicenza, Italy) with a 0.05 mm of layer thickness and bio-compatible photosensitive resin (DS5000, DWS, Vicenza, Italy) (Figure 3); (2) milling technology (Sirona inLab MC X5) using polymethyl methacrylate (PMMA) (Nobil Metal SPA, Villafranca d’Asti, Italy) (Figure 4). 

**Figure 5:**
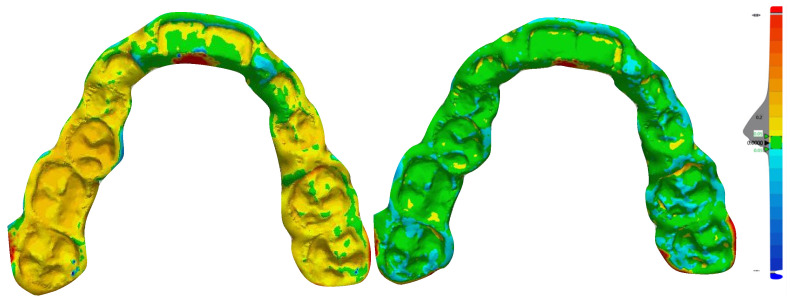
Example of deviation analysis detected after superimposition of the 3D-printed splint (left) and milled splint (right) with the original digital project (evaluation of the trueness).

Both milled and 3D-printed splints were scanned with a high-resolution optical scanner (3Shape A/S, model D500) and the generated .stl files were imported into Geomagic Control X software (3D Systems, version 2018.1.1, 3D Systems, Rock Hill, NC, USA) to calculate the average linear differences (Euclidean distance) between each scanned splint (both prototyped and milled splints) and the superimposed original project (deviation analysis). In particular, the trueness of the splints was calculated with the root to mean square (RMS) values between the original model (digital) and each milled and prototyped splint (digitalized). In this regard, a preliminary point-based superimposition and a definitive surface-based superimposition were performed between the original project and each scanned splint (Figure 5). The range of tolerance set for the analysis was 0.05 mm. According to the generated color map, red-toned colors indicated that the distance values were greater than the positive limits and blue-toned colors indicated that the same values were smaller than the negative limits; by contrast, all values contained in the tolerance range were represented in green. The values of deviation analysis and the volumetric data recorded represented the amount of impression materials included in the digitalized physical splint (airspace). All digital procedures were performed by the same expert operator.

### Statistical Analysis

All the measurements were saved on Microsoft Excel^®^ pages (Microsoft, Redmond, WA, USA) and analyzed using SPSS^®^ version 25 Statistics software (IBM Corporation, 1 New Orchard Road, Armonk, New York, NY, USA). Statistical significance was set at *p* < 0.05. The Shapiro–Wilk test and Levene’s test were used for the assessment of data distribution and equality of variance. The parametric test was used to show eventually relevant changes in data proportions. The trueness of prototyped and milled splints was assessed via the Independent Student’s *t*-test comparing the RMS and the percentage of matching with the CAD project, in accordance with the surface-to-surface analysis. Intraclass correlation coefficient (ICC) was carried to test reliability of the methodology while the Dahlberg’s formula was calculated to investigate the method error.

## 3. Results

Statistically significant differences were detected between prototyped and milled splints in the values of RMS recorded after superimposition with the original digital project (*p* < 0.001) (Table 1). In particular, the milled splints revealed a lower value of RMS relative to the original project (0.20 mm ± 0.018) compared to the prototyped splints (0.31 ± 0.021) (*p* < 0.001). Moreover, the data of the percentage of matching (original digital model as reference) were statistically different between prototyped and milled splints (*p* < 0.001) (Table 2). In particular, the milled splints showed a greater percentage of matching relative to the original project (95.66% ± 1.19) compared to the prototyped splints (66.99 ± 1.38) (*p* < 0.001). Both data of RMS and the percentage of matching reflected higher trueness of those appliances manufactured with milling technology. Concerning the reliability of the methodology, the ICC tests showed no differences between the two intra-operator readings, with excellent correlation indexes ranging from 0.911 to 0.932 for data of deviation analysis. According to Dahlberg’s formula, the random error ranged from 0.004 mm to 0.015 mm.

## 4. Discussion

Occlusal splints are extensively used by dental clinicians and maxillofacial surgeons. The main two limitations of the traditional analogical method for splints generation are (1) the process is extremely complex and time-consuming, (2) specific compensative procedures must be applied in order to counteract the polymerization shrinkage that can alter the fitting of the appliance. Moreover, lab technicians and dentists are exposed to residual monomer vapors and particles produced during the analogical manufacturing method, with a significant increased biological risk and negative effect on the patient’s health [13]. With this notion in mind, continuous advancement in CAD/CAM technology is warmly welcomed in order to expedite the replacement of this technology over conventional analogical procedures.

However, being in the era of digitally-assisted pre-surgery workflow and CAD/CAM dental systems, the establishment of the appropriate manufacturing technology and materials is important for the effectiveness of the surgical splint [4,14]. In orthognathic surgery, surgical splints allow a reliable repositioning of the maxilla or mandible established with the surgical treatment plan. In this regard, any dimensional changes of the 3D-printed splint in comparison with the original digital project can cause errors in bone repositioning with further alterations of the occlusal plane and bite problems (open bite of posterior teeth after surgery) [15]. The trueness issue can also affect the effectiveness of temporary positional guiding devices such as surgical guides for orthodontic miniscrews [16] or dental implants placement [17] and occlusal splints for the treatment of TMJ disorders and for mitigating forces produced during mandibular muscles activity [18].

3D prototyping (additive process) and milling (material removal process) technologies are the main two methods involved in the CAD/CAM system for dental application; however, comparative data of accuracy between both processes in dentistry [15,19,20] are scarce in literature and are limited to single teeth and unilateral mouth [21,22,23] prosthetic rehabilitations or to the accuracy of dental models. In this regard, with the continuous advancements in 3D imaging, is it possible to comparatively estimate morphological and dimensional aspects of anatomic-based objects? In the present study, we used a precise technology involving the surface-to-surface matching technique [24,25,26,27,28] that allows the superimposition of 3D objects and the evaluation of point-to-point surface distances between the registered models. The analysis was enriched by the 3D color-coded map showing the morphological differences between the registered structures in a color-based scale by setting specific levels of tolerance. By this method, we were able to detect if the printed splints moved away from the original digital project and to report the consistency/inconsistency data as a percentage of matching/mismatching between the original digital splint and the scanned-printed splint.

Data of deviation analysis confirmed that the scanned-milled surgical splints had greater trueness compared to the scanned-prototyped splints, as suggested by the differences in the percentage of matching (3D Project/Milled matching = 95.66% ± 1.19; 3D Project/Prototyped matching = 66.99% ± 1.38) and in the values of RMS (3D Project/Milled matching = 0.20 mm ± 0.018; 3D Project/Prototyped matching = 0.31 mm ± 0.021) with the master digital project. Although previous evidence confirmed that milling technology is more accurate than prototyping process [12], the differences of trueness between milled and prototyped splints (almost 30%) found in this study could have been emphasized by the digitalization process of physical splints. A recent study showed an increment of the linear vertical and transverse dimension of milled mock-ups; instead, the 3D-printed mock-ups had only a small increment of transverse measurements, in comparison with the master digital project. Nevertheless, these findings were balanced in the virtual environment where scanned-milled mock-ups showed greater trueness while the prototyped mock-ups showed some percentage of underestimation. In this regard, it was previously found that after CAD/CAM digitization, the same measurements performed on the virtual environment can be reduced [29], probably due to reductive algorithmic computation. Accordingly, it is possible that this intrinsic bias of the digitalization process may have slightly overestimated the difference of trueness recorded in the present study between milled and prototyped surgical splints.

Previous evidence suggests that milling technology may present some biases when the production of thin objects is required. It has been found that the fabrication of mock-ups or veneers may be altered since the cutting tool (the bur) may not sufficiently penetrate the block of resin. This would change the increment of the final measurement of the object, causing potential fitting issues [29,30,31]. Similarly, it is possible that some inaccuracy could be obtained in the occlusal surface of the splint, which is characterized by thin anatomical grooves reflecting thin occlusal interdigitations. However, the surgical splint is thick enough to allow an effective 3D movement of the bur, and this would explain why we found greater trueness by using milling technology in the present study. In this respect, the present study provides new evidence and new unanswered questions that need further validation in new studies.

Despite the two main advantages of 3D printing over a milling machine, i.e., efficiency and the least amount of material necessary to fabricate dental manufacture [32,33,34], the present findings suggest that the surgical splints produced with milling technology are more accurate than those obtained with prototyping and that further advancement in 3D technology is required in order for it to be considered a valid alternative to a milling machine. However, these findings should be considered with caution since they lack clinical response in terms of fitting and tolerance and further studies are required to assess this aspect.

It must be underlined that the present study was focused on testing ex-vivo the accuracy of 3D printing and milling technologies for the generation of the occlusal splint, without a clinical validation based on the fitting of the appliance after surgery. In this regard, we are conscious that the effectiveness of orthognathic surgery is influenced by the diagnosis, treatment plan strategy, and the technical skills of the operator.

### Study Limitations

The study was limited to small sample size as well as to a single device for each process used to generate the physical splints, i.e., prototyping and milling technologies. Considering that 3D printing technology is spreading in clinical dentistry, further studies are required to test the accuracy of different 3D printing technologies in generating specific appliances, included surgical splints.

Furthermore, the layer thickness for the 3D printing process was set at 0.05 mm in this study, which increased the printing time and cost compared to a layer thickness of 0.010 mm. In this regard, future studies could investigate the prototyping process of surgical splints set at different layer thicknesses, comparing not only the accuracy but also the setting parameter to find the best balance between accuracy, tolerance, and costs.

## 5. Conclusions

The findings of the present investigation would suggest that surgical splints are more accurate when produced by milling technology, according to the greater percentage of matching found in relation to the original digital project. Clinicians should be aware of this when referring to the lab technician for the construction of the appliance.

## Figures and Tables

**Table 1:** Comparative data of deviation analysis (root to mean square (RMS) values) between milled and prototyped splints related to the 3D project. *p* value based on Student’s *t* test and set at *p* < 0.05.

Matching Percentage			
Type	n. Total	Mean ±SD	Significance (*p*)
Prototyped	10	0.20 mm ± 0.018	*p* < 0.001
Milled	10	0.31 mm ± 0.021	

**Table 2:** Comparative data of percentage of matching between milled and prototyped splints related to the 3D project. *p* value based on Student’s *t* test and set at *p* < 0.05.

Matching Percentage			
Type	n. Total	Mean ±SD	Significance (*p*)
Prototyped	10	95.66 ± 1.19	*p* < 0.001
Milled	10	66.99 ± 1.38	
